# Research on the Method of Depression Detection by Single-Channel Electroencephalography Sensor

**DOI:** 10.3389/fpsyg.2022.850159

**Published:** 2022-07-13

**Authors:** Xue Lei, Weidong Ji, Jingzhou Guo, Xiaoyue Wu, Huilin Wang, Lina Zhu, Liang Chen

**Affiliations:** ^1^School of Business, East China University of Science and Technology, Shanghai, China; ^2^Mental Health Center, East China Normal University, Shanghai, China; ^3^Shanghai Changning Mental Health Center, Shanghai, China; ^4^Shanghai Fujia Cultural Development Co., Ltd., Shanghai, China

**Keywords:** depression detection, state depression, EEG sensor, PHQ-9, HAM-D, house-tree-person (HTP) drawing test

## Abstract

Depression is a common mental health illness worldwide that affects our quality of life and ability to work. Although prior research has used EEG signals to increase the accuracy to identify depression, the rates of underdiagnosis remain high, and novel methods are required to identify depression. In this study, we built a model based on single-channel, dry-electrode EEG sensor technology to detect state depression, which measures the intensity of depressive feelings and cognitions at a particular time. To test the accuracy of our model, we compared the results of our model with other commonly used methods for depression diagnosis, including the PHQ-9, Hamilton Depression Rating Scale (HAM-D), and House-Tree-Person (HTP) drawing test, in three different studies. In study 1, we compared the results of our model with PHQ-9 in a sample of 158 senior high students. The results showed that the consistency rate of the two methods was 61.4%. In study 2, the results of our model were compared with HAM-D among 71 adults. We found that the consistency rate of state-depression identification by the two methods was 63.38% when a HAM-D score above 7 was considered depression, while the consistency rate increased to 83.10% when subjects showed at least one depressive symptom (including depressed mood, guilt, suicide, lack of interest, retardation). In study 3, 68 adults participated in the study, and the results revealed that the consistency rate of our model and HTP drawing test was 91.2%. The results showed that our model is an effective means to identify state depression. Our study demonstrates that using our model, people with state depression could be identified in a timely manner and receive interventions or treatments, which may be helpful for the early detection of depression.

## Introduction

Depression is the most prevalent mental health disorder and a leading cause of disability worldwide. Globally, more than 300 million people in the world are living with depression, and it has increased 50% in incident cases from 1990 to 2017 (Liu et al., 2020; Hobden et al., 2021). People with depression may greatly suffer and function poorly at work, at school and in the family. Because depression can be long-lasting or recurrent and lead to suicide (Stringaris, 2017; Liu et al., 2020), the World Health Organization (WHO) ranks it as a major contributor to the global burden of disease (Bhatti et al., 2016). In addition, patients with major depressive disorder have increased risks of developing cardiovascular disease and increased morbidity and mortality (Seligman and Nemeroff, 2015; Luo et al., 2018). Although depression incurs huge psychological and physical harm to human beings, people who are depressed are often not accurately identified and treated. As a result, identifying and diagnosing patients with depression early and giving timely treatment are important issues to solve. Early detection of depressive tendencies and mild depression is conducive to timely and active intervention, which can avoid further aggravation of depression (Dong et al., 2020). Therefore, new methods are required to accurately assess depression.

Depression is considered a mood disorder that lasts for at least 2 weeks, with the presence of depressed mood and loss of pleasure being the core features (Su et al., 2021). The severity of symptoms of depression varies from feeling sad or gloomy in a relatively short period of time to extreme hopelessness, despair, extreme guilt, and thoughts of death that can lead to suicide (Spielberger and Reheiser, 2009). Obviously, depression is a complex and multifaceted syndrome with many potential dimensions. In this study, we focus on state depression, which is defined as the intensity of depressive feelings and cognitions at a particular time (Spielberger and Reheiser, 2009).

In the past, depression is hard to precisely diagnose mainly because of the subjectivity of patients to answer the psychological evaluation. With the development of electroencephalography (EEG), scientists began to use it as a tool to diagnose many neurological and psychological diseases, such as depression. EEG activity reflects the temporal aggregation of synchronous activity of millions of spatially aligned cortical neurons. The most deliberated waveforms include delta (0.5–4 Hz), theta (4–7 Hz), alpha (8–12 Hz), sigma (12–16 Hz), and beta (13–30 Hz). Compared to the traditional diagnose evaluation using questionnaires, EEG biomarkers could help diagnose the depression in a more objective way. Extensive studies have explored the use of EEG to find depression biomarkers for diagnostic purposes (de Aguiar Neto and Rosa, 2019; Mahato and Paul, 2020). According to the research, the spectral coupling between the delta and beta oscillations has been proven to be related to social anxiety (Miskovic et al., 2011). And over recent decades, many researchers have attempted to detect Schizophrenia from EEG signals (Kim et al., 2015; Johannesen et al., 2016). Recently, researchers have been developing an automatic Schizophrenia identification scheme using EEG signals that can eradicate the aforementioned problems and support clinicians and researchers (Siuly et al., 2020).

Linear features including Band Power and Alpha Asymmetry have been studied for diagnosing depression. For instance, gamma and theta band have good depression diagnostic capabilities (Mohammadi et al., 2015; Fitzgerald and Watson, 2018), whereas alpha asymmetry may predict specific symptoms and treatment outcome (Nelson et al., 2018), although for diagnostic purposes it may not be suitable (van der Vinne et al., 2017). Compared with linear feature measures, non-linear methods such as Detrended Fluctuation Analysis (DFA), Higuchi’s Fractal Dimension (HFD), Lempel-Ziv Complexity (LZC) could obtain additional information for analyzing EEG signal features (Hosseinifard et al., 2013; Mumtaz et al., 2015; Bachmann et al., 2018). Considering signal complexity, HFD appears to be higher in depressed brains (Mahato and Paul, 2019). Besides, network-based features (e.g., Cluster Coefficient, Zhang et al., 2018) and evoked potentials (e.g., Late Positive Potential, Grunewald et al., 2019) have been studied to be the potential depression biomarkers. Among these research, recent studies utilizing machine learning techniques is showing a great success in the automatic detection of depression (Hosseinifard et al., 2013; Lin et al., 2018). For instance, machine learning algorithms combining support vector machine (SVM) and convolutional showed a high accuracy for depression recognition (Li et al., 2019).

However, most of the previous studies detected depression based on analyzing multichannel EEG signals. Although EEG sensors in most previous studies can precisely record brain activities with proper preparation, their usage is only limited to the clinical and laboratory setups because the sensors are multichannel (such as 256 channels), use wet electrodes and transmit data via a set of wires, which demand longer time for preparation and offer lower usability (Patel et al., 2017). R To increase common use, recent developments in techniques simplified these multichannel EEG devices (Ni et al., 2020). Therefore, in this study a wearable Bluetooth headset EEG acquisition device (NeuroSky headset) is used, which is easy to wear and computationally efficient (Bhatti et al., 2016). Studies considering depression recognition based on single channel EEG signal analysis are relatively limited. Spectral asymmetry index (SASI) was successfully applied for detection of depression employing single channel EEG (Bachmann et al., 2017). Depression recognition based on single channel EEG signal is a promising trend.

By relying on attention and meditation signals recorded from the MindWave mobile headset, a single-channel and dry-electrode EEG product produced by the NeuroSky company, we built a model to detect state depression. Many researchers are interested in NeuroSky’s MindWave mobile headset because of its higher usability at a significantly lower cost and the ability to conduct research in informal environments such as schools and homes (Sezer et al., 2015; Ni et al., 2020).

NeuroSky’s MindWave mobile headset was developed as a biosensor to record EEG data signals and can be used to detect the electric activities of the brain in a state of attention and relaxation (Sezer et al., 2015; Ni et al., 2020). The MindWave mobile headset comprises an TGAM EEG acquisition module (EEG acquisition point), a forehead electrode (reference point), an ear clip electrode (grounding circuit), a Bluetooth transmitting module, a Bluetooth receiving module, as well as a computer connected to the device (Zhang et al., 2019). Since the device uses a dry-sensor, it requires no saline or gel in order to ensure proper connectivity with the surface of the forehead and noise-free EEG signals. Contact with the dry sensor electrode is achieved by the pressure of the electrode against the subject’s forehead and held in place by the headset. The sampling rate is 512 Hz. The headset is easy to wear on head while the sensor is placed on forehead at FP1 location (according to 10/20 EEG placement system) and a reference electrode is connected to the ear lobe to complete the circuit. The main benefit of this headset was the ease in data acquisition setup and wearability. The headset used Bluetooth to transmit recorded data to the hardware host for analysis and hence provided mobility to the end user (Bhatti et al., 2016). The NeuroSky company has conducted benchmark tests by comparing EEG signals measured by the NeuroSky headset with signals from the Biopac system, a well-known wet electrode EEG system widely used in medical and research applications (NeuroSky, 2009).

The TGAM module is used by the MindWave mobile headset to process and output the brain wave spectrum, original brain wave, EEG signal quality, and two NeuroSky eSense parameters: attention and meditation detection (see Supplementary Appendix A for more details about the TGAM module). The device quantifies the psychological state of the subjects as attention and meditation values (Sezer et al., 2015; Ni et al., 2020). The reliability of the attention and meditation meters have been supported by previous studies (Rebolledo-Mendez, 2009; Crowley et al., 2010). The MindWave mobile headset also has noise-filtering technology to eliminate interference from daily living environments or other electronic equipment.

In this study, using “attention’’ and “meditation^1^” indices from NeuroSky’s MindWave mobile headset, we discovered an M-shaped brainwave that accompanies the occurrence of depressive emotions and thus built a model to detect state depression. We hypothesized that our brainwave model is an effective means to identify state depression, i.e., transient states of depressive thoughts/emotions. This method can assist in detecting and correctly diagnosing depression early in a large population, therefore individuals with depression can receive timely interventions or treatments.

Specifically, to examine whether our model can correctly identify state depression, we compared the results with other commonly used methods for depression detection, such as the PHQ-9, Hamilton Depression Rating Scale (HAM-D), and House-Tree-Person (HTP) drawing test, a type of psychological projective technique (Buck, 1948; Chen and Xu, 2008).

## Materials and Methods

Below we provide detailed information for the pilot study and the following three studies. First, in the pilot study, we discovered an M-shaped pattern in EEG brain waves during a psychological counseling intervention for depression and developed a model for state depression detection. To confirm this model was a reliable measure for state depression, we further conducted study 1–3 in samples of senior high students, adults who were seeking psychological assistance, and healthy adults. In study 1, we compared the results of our model with PHQ-9. The results showed that the consistency rate of the two methods was 61.4% among 158 subjects. In study 2, the results of our model were compared with HAM-D among 71 subjects. We found that the consistency rate of state-depression identification by the two methods was 63.38% when a HAM-D score above 7 was considered depression, while the consistency rate increased to 83.10% when subjects showed at least one depressive symptom (including depressed mood, guilt, suicide, lack of interest, retardation). In study 3, 68 subjects participated in the study, and the results revealed that the consistency rate of our model and HTP drawing test was 91.2%.

### Pilot Study Discovering the M-Shaped Brainwaves

#### Measurement Devices

NeuroSky’s MindWave mobile headsets were adopted in all the studies. This is a commercial EEG device to measure brain waves. Prior research has shown that brain waves have a frequency value of 0–100 Hz (Mostow et al., 2011) and are grouped into five basic bands: delta (0–3.5 Hz), theta (4–7 Hz), alpha (8–12 Hz), beta (13–30 Hz), and gamma (>30 Hz) (Neurosky, 2011; Sałabun, 2014; Sezer et al., 2015). There is also a direct correlation between frequency and brain activity (Sezer et al., 2015). In our study, the device captures brain waves and produces an original value. The results of the data analysis were evaluated with the manufacturer’s eSense Metric, and the value of attention and meditation was 0–100 (Neurosky, 2011; Sezer et al., 2015). Previous studies have tested the reliability of NeuroSky’s MindWave headsets in measuring attention and meditation (Sezer et al., 2015) and confirmed that the device had good measurement accuracy (Ni et al., 2020).

#### Procedures and Findings

**FIGURE 1:**
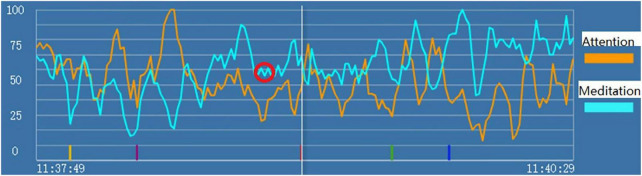
“Attention” (the orange line) and “meditation” (the blue line) signals during psychological counseling intervention for depression of Subject A1. The red circle indicates the markers of state depression.

In the initial exploration stage, we recorded the brainwaves of subjects during the entire process of psychological consultation using the headsets. We found that when the subjects were depressed, an M-shaped pattern appeared in their brainwaves of “attention” and/or “meditation” signals. Specifically, we first found an M-shaped pattern when subject A1 was receiving a psychological counseling intervention for depression, and such patterns as circled in red in Figure 1 repeatedly appeared at multiple time points in the attention and meditation signals when subject 1 showed symptoms of depression like mentioning helpless feelings or thoughts of committing suicide. Then, we verified this phenomenon in various subjects. For example, subject A2 communicated with one of our researchers for the first time, and she cried and talked about all types of unpleasant experiences and problems in her family. During this process, the M-shaped pattern also appeared in her attention and meditation signals.

Therefore, we hypothesized that when subjects have state depression, M-shaped patterns appear in their attention and/or meditation signals. Building on this phenomenon, we developed a specific model to identify state depression based on the brainwave, with detailed criteria to classify the M-shaped patterns. More details about the model are described in our patent (patent number 202110609863.9).

#### Discussion

In the initial exploration stage, we developed a model to detect state depression based on the attention and mediation signal produced by a single-channel EEG headset. In order to verify the accuracy of our model, in Study 1–3, we compared the results of our model with other methods to diagnose depression, including the PHQ-9, Hamilton Depression Rating Scale (HAM-D), and House-Tree-Person (HTP) drawing test (Buck, 1948; Chen and Xu, 2008; Gu et al., 2020).

### Study 1 Electroencephalography Model of State Depression and Patient Health Questionnaire-9

#### Materials and Methods

##### Subjects

We recruited 161 senior high school students from a boarding high school in Shanghai. Among these, three students did not have valid brainwave data, which led to a final sample of 158 subjects. This study was conducted with approval from the Institutional Review Board (IRB) at the second author’s institute under strict supervisions to protect subjects’ privacy and participants have the right to end the experiment at any time. Following IRB instructions, consent forms were obtained from the students’ parents before the experiment.

##### Measures

PHQ-9 is a self-report questionnaire that has been used widely to screen depression in primary care (Zuithoff et al., 2010; Thekkumpurath et al., 2011; Zhang et al., 2013; Wang et al., 2014). The PHQ-9 (Supplementary Appendix B) is the 9-item depression module from the full PHQ, which is a self-administered version of the PRIME-MD (Kroenke and Spitzer, 2002). PHQ-9 satisfies five practical considerations: brevity, easy to score, self-administered, multi-purpose, and in the public domain (Kroenke, 2012). The reliability and validity of PHQ-9 have been examined in prior research (Kroenke et al., 2001; Zhang et al., 2013; Wang et al., 2014). It has been translated into more than 80 languages and is widely used worldwide in different populations (Wang et al., 2014). Subjects were asked to rate how they felt within the past 2 weeks. Each question was scored from 0 to 3 (0 = not at all; 1 = several days; 2 = more than half of all the days; 3 = nearly every day). Scoring 0–4 on PHQ-9 indicates minimal/no depression, 5–9 mild depression, 10–14 moderate depression, 15–19 moderately severe depression, and 20–27 severe depression (Fiest et al., 2016; Gill et al., 2017).

##### Procedures

Subjects completed the self-rating PHQ-9 after collecting the brainwave data. Specifically, first, before recording the brainwave data, we asked the subjects whether they had practiced yoga, meditation, tai chi, qigong and other attention exercises before. If they had practiced these exercises, they were required not to practice while their eyes were closed, as engaging in these can alter the baseline brainwave pattern. Then, they were asked to wear our single-channel EEG device, close their eyes and remain in a resting state for 3 min. During this process, we asked them to try to relax and not think about anything. After 3 min, the subjects were asked to remove the device and complete the self-rating PHQ-9 questionnaires.

#### Data Analysis

First, we collected and analyzed the brainwave data of the subjects in the resting state. To avoid subjective bias, we invited two researchers blinded to the subjects’ PHQ-9 scores to independently identify the frequency and severity of state depression from the attention and meditation signals, based on the criteria developed in the pilot study. Any disagreements were all resolved through discussion and refinement of the criteria. Then, we compared the results with PHQ-9 questionnaires. Subjects were classified as depressed or non-depressed based on their PHQ-9 scores (Zhang et al., 2013). The PHQ-9 scores range from 0 to 27, since each of the nine items can be scored from 0 (“not at all”) to 3 (“nearly every day”). Based on commonly used criteria (Fiest et al., 2016; Gill et al., 2017), we chose a cutoff score of 5 as indicating state depression, which is lower than clinical diagnosis of depression.

#### Study 1 Results and Discussion

The results showed that the overall consistency rate between our model and PHQ-9 was 61.4%^2^. The results of the identification of state depression by our model and PHQ-9 are shown in Table 1. Our model identified more depressed individuals compared to PHQ-9 (98 vs. 81). Of note, our EEG model measures transient or current depressive emotions while PHQ-9 measures depressive emotions during the past 2 weeks. Thus, the difference between PHQ-9 results and our brain wave model may be caused by difference in time range. In addition, people can lie when completing self-report diagnostic questionnaires. Therefore, the difference in results may also imply that some of the subjects did not report honestly in the questionnaire or did not have a clear understanding of their inner feelings.

**TABLE 1:** Comparison of results between our model and PHQ-9, HAM-D, HTP, respectively.

Comparative depression measurement	Total number	Identified state-depression by our model	Identified no state-depression by our model	Consistency rate
**PHQ-9 (above 4)**				
*Identified state-depression*	81	59	22	61.4%
*Identified no state-depression*	77	39	38	
**HAM-D (above 7)**				
*Identified state-depression*	43	40	3	63.38%
*Identified no state-depression*	28	23	5	
**HAM-D (above 1)**				
*Identified state-depression*	57	54	3	83.10%
*Identified no state-depression*	14	9	5	
**HTP test**				
*Identified state-depression*	53	51	2	91.2%
*Identified no state-depression*	15	4	11	

To overcome the limitations of self-report depression questionnaires, we adopted a more rigorous and other-rated depression diagnostic method in Study 2.

### Study 2 Electroencephalography Model of State Depression and Hamilton Depression Rating Scale

The 17-item Hamilton Depression Rating Scale (HAM-D) is one of the most widely used measures of depression (Bech, 2015; Bech et al., 2015; Lee et al., 2017) and has been used as a “gold standard” instrument to assess the severity of depression in clinical studies (McIntyre et al., 2002). Thus, we selected HAM-D as the instrument in Study 2 to compare with our EEG model results.

#### Materials and Methods

##### Subjects

In Study 2, we recruited 73 adults aged 18–60 years who were suffering from various emotional disturbances and seeking psychological assistance. The sample size was decided prior to the experiments with an effort to recruit as many subjects as possible given the resource limitation within around 2 weeks. Two subjects did not have valid brainwave data, resulting in 71 subjects in our final sample. This study was conducted with approval from the Ethics Committee of the second author’s institute. Besides, consent forms were collected from subjects before the experiment.

##### Procedures

Before the experiment, subjects were asked about relevant medical history, medication history, and basic information such as age and gender. Then, subjects were informed of the purpose of the study and were required to sign an informed consent form. Next, in the experiment, the subjects were required to wear the MindWave mobile headset. Two trained researchers, both of whom had a background in clinical psychology, independently scored the subjects based on the depression part of the Hamilton Questionnaire interview and recorded the brain waves of the subjects during the interview.

##### Measures

The HAM-D17 consists of 17 items, nine of which are scored on a five-category Likert scale (0–4) and the remaining eight on a three-category Likert scale (0–2), giving a total score ranging from 0 to 52 (Licht et al., 2005). The 17 items of HAM-D are shown in Supplementary Appendix C (McIntyre et al., 2002). According to commonly accepted criteria (Hamilton, 1960; Rashid et al., 2021), scoring 0–7 on the 17-item HAM-D is considered as being normal, 8–16 suggests mild depression, 17–23 implies moderate depression, and over 24 is indicative of severe depression.

#### Data Analysis

The HAM-D questionnaires were rated by two trained clinical researchers during the interview who were blinded to the brainwave results. The percent of agreement of the two researchers was 97.0% when considering scores above 7 as having state depression. As for the brainwave data, two researchers on our research team independently identified the frequency and severity of state depression from the attention and meditation signals based on the updated criteria from Study 1. Any discrepancies were resolved through discussion.

#### Study 2 Results and Discussion

According to prior research, full remission of depression is defined as an HAM-D_17_ of 7 or less (Frank et al., 1991). Thus, an HAM-D score above 7 was considered a sign of depression, resulting a consistency rate of 63.38% between our brainwave model and HAM-D^3^. The detailed results of the identification of state depression by our model and HAM-D are shown in Table 1.

Moreover, if we also consider the items with some depressive characteristics (including depressed mood, guilt, suicide, work and interest, retardation) above 1 as early stages of depression, then the consistency rate of the two methods is 83.10% (see Table 1). In particular, we found two subjects in Study 2 who had severe depressive emotions or thoughts during the experiment, such as crying and conveying suicidal thoughts. During these moments, the M-shaped patterns simultaneously appeared in their brainwaves.

Therefore, to better capture the emergence of depressive emotions/thoughts and to demonstrate the simultaneity of our brainwave model and these transient feelings, we adopted the house-tree-person drawing test in Study 3.

### Study 3 Electroencephalography Model of State Depression and House-Tree-Person Drawing Test

#### Materials and Methods

##### Measures

The house–tree–person drawing test is a type of projective technique that is designed to obtain information concerning the sensitivity, maturity, and integration of a subject’s personality and the interaction of that personality with its environment (Buck, 1948). The HTP drawing test includes two phases. The first phase is drawing, which is non-verbal, creative, and almost completely unstructured. The second phase is describing and interpreting the drawing, which is verbal, apperceptive, and more formally structured (Buck, 1948). Then, the subject’s responses are analyzed in diverse manners, such as what was said and on which aspect of the drawing was focused. The results are based on psychodynamic interpretation of the details of the drawing, such as size, shape, and complexity (Gu et al., 2020). Using this technique, subjects can freely express their thoughts and feelings that they find difficult to articulate (Donoghue, 2000; Gu et al., 2020); then, the researcher can enter the subjects’ private worlds to uncover their inner perspectives, hidden emotions and internal conflicts (Donoghue, 2000; Gu et al., 2020). According to the literature, the House–Tree–Person drawing test is reliable and valid (Chen and Xu, 2008). Researchers have used it to diagnose neurosis and depression and found certain indicators that are reliable markers for these mental disorders (Kan et al., 2011; Cai et al., 2012; Gu et al., 2020). In addition, the House–Tree–Person drawing test is very helpful in diagnosing emotional disorders (Inadomi et al., 2003), and it has been used in emotional tests, psychological screening, and post-disaster relief programs (Chen and Xu, 2008; Gu et al., 2020).

##### Subjects

This research was approved by the Institutional Review Board at the first author’s university. Consent forms were also collected from subjects before the experiment. The sample size was decided prior to the experiments with an effort to recruit as many subjects as possible given the resource limitation within around 2 weeks. We recruited 69 volunteers aged 18–60, who were healthy, had no neurological or psychiatric illness, did not suffer from emotional disturbances or who wanted to seek psychological assistance. Because one subject did not have valid brainwave data, the final sample included 68 subjects. Among the 68 subjects, 44.1% were male, and the average age was 31.3 years old. Before the experiment, the subjects were informed about the procedure of the study and agreed to participate in the study.

##### Procedures

We followed prior research on the House-Tree-Person (HTP) drawing test to make the experimental procedures more standardized (Buck, 1948; Oster and Crone, 2004; Moschini, 2005; Chen and Xu, 2008). The materials for HTP tests include a piece of A4 paper, a 2B pencil, an eraser, and a ruler (if the subject asked for). Subjects were instructed to draw following the rules: (1) Please draw at least three things on your paper, including a house, a tree, and a person. (2) The drawing does not need to be aesthetically pleasant, but you have to draw carefully and draw whatever you want. (3) After completing the drawing, write down your name, age, occupation, and education level at the bottom of the paper. During the drawing test, we simultaneously collected the brainwave data of the subjects.

Although there is no limit on drawing time, it is assumed that 5–30 min is the normal range, and below or exceeding this range is of analytical significance. After the drawing test, an interview was conducted while a psychological counselor interpreted to each participant the type of psychological or emotional states reflected by the drawing. The participants were asked if they agreed on the counselor’s interpretations.

#### Data Analysis

**FIGURE 2:**
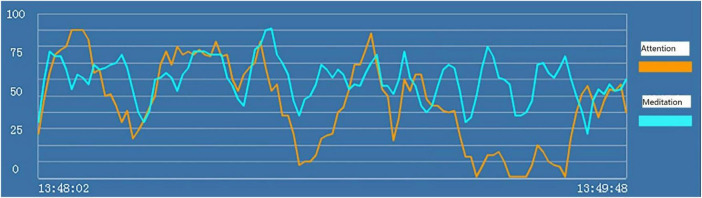
“Attention” (the orange line) and “meditation” (the blue line) signals in the HTP drawing test for subject A3 who did not have state depression.

**FIGURE 3:**
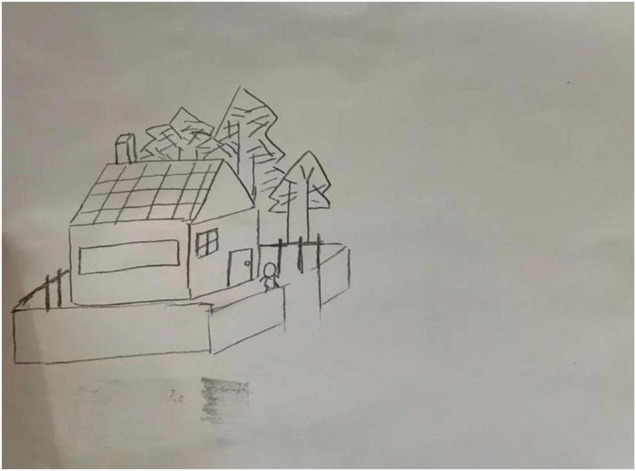
A picture drawn by subject A3 who did not have state depression.

For each subject, we recorded the brainwave data during the HTP drawing test (as shown in Figure 2), a picture (as shown in Figure 3) and a transcript of the interview. First, to analyze the brainwave data, same as before, we invited two researchers to independently and simultaneously identify the frequency and severity of state depression from the attention and meditation signals in each part. With regard to analyzing data from the drawing test, each drawing was inspected and rated by a team of clinical researchers based on an agreed-upon HTP diagnostic classification table, which includes a series of standardized indicators such as the size of the person, symmetry and position of the tree. The team consists of five senior psychological counselors with rich experience in the application of the HTP drawing test.

#### Study 3 Results and Discussion

**FIGURE 4:**
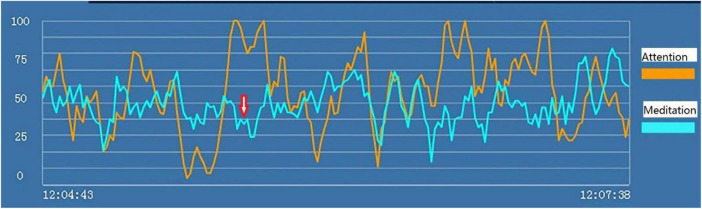
“Attention” (the orange line) and “meditation” (the blue line) signals in the HTP drawing test for subject A4 who had state depression. The red arrow indicates the markers of state depression.

**FIGURE 5:**
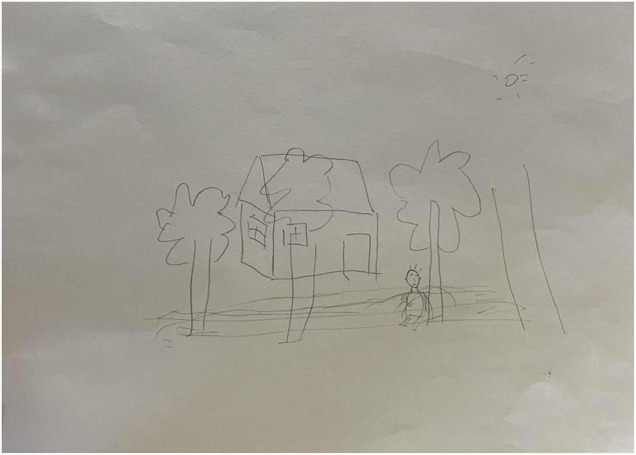
A picture drawn by subject A4 who had state depression.

Examples of the brainwave data and pictures of two subjects are displayed. Figures 2, 3 show the brainwave data and pictures in the HTP drawing test of subject A3 who did not have state depression. Figures 4, 5 show the brainwave data and pictures in the HTP drawing test of subject A4 who had state depression. According to the psychological interpretation of the drawing in Figure 5, it mainly presents two depressive characteristics: the brush strokes are shallow, which implies that subject A4 lacks strength and self-confidence; the person in the picture is leaning against a tree, which shows a strong sense of powerlessness.

Study 3 results revealed that the consistency rate of our brainwave model and HTP drawing test was 91.2%. The detailed results of the identification of state depression by our model and HTP are shown in Table 1.

To better compare the results of the two methods, we provided specific examples in Table 2 to show subjects’ depressive thoughts/feelings and the corresponding depressive EEG signals. Particularly, for each subject, we compared the time of state depression detected by the single-channel EEG sensor and the corresponding psychological interpretation of the drawing in the HTP drawing test. All subjects endorsed the psychological interpretations. The results again confirmed that our model can effectively identify state depression.

**TABLE 2:** Examples of the depressive thoughts/feelings of subjects and the corresponding depressive EEG signal.

Subject	The time of state-depression detected by the single-channel EEG sensor	The psychological interpretation of HTP drawing test simultaneously
Subject 1	1′24″∼1′28″	Subject 1 was hesitating, which showed his inner struggle as he was trying to find a way out his problems.
Subject 2	(1) 00′53″∼00′57″ (2) 1′23″∼1′27″ (3) 2′59″∼3′03″ and 3′16″∼3′20″	(1) Subject 2 felt pressure from all aspects in his lives. (2) Subject 2 had anxious conflicts about his inner self-cognition. (3) Subject 2 did not have clear knowledge about himself, and he had hidden anxiety and depression.
Subject 3	8′04″∼8′08″	Subject 3 was anxious and worried about the future and did not know how to move on.
Subject 4	(1) 00′58∼1′02″ (2) 6′∼6′04″	(1) Subject 4 had some inner conflicts with family-related matters. She was unwilling to communicate and avoided interpersonal communication. (2) Subject 4 felt anxious and stressed in career and social relationships, and she also had strong psychological defense, self-enclosure and self-protection.
Subject 5	(1) 2′18″∼2′22″ (2) 3′14∼3′18	(1) Subject 5 wanted to find a place to shelter from wind and rain and rest, which caused some negative feelings. She also felt a sense of powerlessness in her heart. (2) Subject 5 drew very small human figures in the picture, which implied weak personal inner strength.

## General Discussion

Results across three studies showed that state depression can be detected using our model building on the “attention” and “meditation” brainwaves produced by NeuroSky’s MindWave mobile headsets. When users of the MindWave mobile headset were in a state of depression, an M-shaped pattern appeared in attention and/or meditation signals. We compared the results of our model with other methods to diagnose depression, including PHQ-9, HAM-D, and HTP drawing test, and confirmed the validity of our model.

In addition, to explain the discrepancies in some results, we followed-up with two subjects in Study 2, whose results from the HAM-D questionnaire and the brainwave test were quite different. For example, the brain waves of one subject showed significant state depression in our experiment, but the HAM-D questionnaire did not identify depression. We invited the subject to take another test 2 weeks later, and the severity of state depression in the brain wave test was significantly relieved. We asked the subject and learned that the subject was preparing for job applications a few days before the first test. At that time, she did not know how to deal with the stress and was slightly lost. However, she did not realize her psychological problems during the HAM-D questionnaire interview. Later, with the help of her classmates, she developed plans and began to submit resumes. Therefore, when we tested her again, her depression and anxiety symptoms were significantly reduced. This result further demonstrated that our model of the brainwave test is a sensitive measure of transient state depression.

Depression detection has gained much attention in psychological research, but the detection of state depression using EEG signals has been rarely studied. The key contributions and strengths of this study are three-fold. First, the results of this study can be used for objective, accurate, and rapid diagnoses of depression to help detect depression early and to monitor the stage of depression continuously. Although prior studies have used EEG signals to increase the accuracy to identify depression patients, the rates of underdiagnosis and undertreatment remain high, and there are few methods for early detection of depression. Identifying patients at an earlier stage in their mental illness creates more opportunities for treatments and appropriate care resources. Using the model in our study, we identified depression in a timely and accurate manner. Patients with state depression can thus acquire timely treatment to avoid further aggravation of depression (Dong et al., 2020).

Second, our model is based on single-channel, dry-electrode EEG sensor technology, which largely simplified signals from traditional multichannel EEG sensors. Thus, it is available for common and popular use and may also be helpful for large-scale screening of depression (Ni et al., 2020). Previous research mostly used multichannel EEG sensors to precisely record brain activities and identify depression patients (Mahato and Paul, 2019). But the usage of multichannel EEG sensors was largely limited to clinical and laboratory setups because of the longer preparation time and lower usability. The recent development of the single-channel EEG sensor features higher usability at a significantly lower cost, increasing the possibility of conducting studies in informal environments such as schools and homes. In this case, more people can use our model based on the single-channel EEG device to detect their state of state depression and identify depression at an earlier stage.

Third, the adoption of a multi-measurement method to compare our brainwave model with three currently commonly used diagnoses of depression (PHQ-9, HAM-D, HTP test) greatly increased the validity of our model, given that one method may not be accurate enough to identify depression. Besides, applying the method across three samples ranging from high school students to adults, and including both healthy people and depressed patients, also enhanced the generalizability of our brainwave model to a large population.

Despite the strengths, the current study is not without limitations. Firstly, due to the scope of the study, we were not able to examine the specific mechanism of the relationship between our model of M-shaped patterns and depression. Future study can further explore the underlying mechanism in EEG wave. Moreover, it is a possible direction to analyze the pathogenesis of depression by adopting various analytical methods and techniques and integrating multiple factors into a unified model for analysis to achieve a more objective, comprehensive, scientific and accurate diagnosis and treatment goal.

Secondly, although we included three studies and expanded our effort to recruit more subjects, the sample sizes may not be large enough to generalize to other samples. It would be meaningful for future studies to increase the sample size and conduct tests in a larger population. In addition, researchers can further explore different models to identify other emotional states, such as anxiety, based on “attention” and “meditation” indices from the single-channel EEG sensor. Other efficient methods of processing EEG data will be investigated on a larger database in the future.

Lastly, as a first study using a single-channel, dry-electrode EEG sensor to measure depressive emotions, depressive emotions were only measured once for each subject. But future research can further explore the reliability of state depression and determine whether multiple measurements can improve the accuracy of depression detection. Besides, future studies can identify the factors that promote or inhibit the production of state depression, such as thinking about happy or depressed events, which may help to effectively improve depression symptoms.

In sum, we introduced an EEG brainwave model to identify state depression with a consistency rate from 61.4 to 91.2%, which helps the quick diagnosis and assistance of depression at an early stage. More importantly, research on emotion recognition and adjustment methods based on EEG has very important practical significance and application prospects in the fields of medical health, work life, and human-computer interaction. The root of emotion is in the brain. Electroencephalogram (EEG) can reflect various activity states of the brain and characterize its neural activity during emotional changes. The use of single-channel, dry-electrode EEG sensors to study the recognition and regulation of emotional states and to explore the inner neural mechanism likely would continue to progress in the future.

## Data Availability Statement

The raw data supporting the conclusions of this article will be made available by the authors, without undue reservation.

## Ethics Statement

The studies involving human participants were reviewed and approved by the Ethics Committee at School of Business, East China University of Science and Technology, as well as Shanghai Changning Mental Health Center. Written informed consent to participate in this study was provided by the participants or participants’ legal guardian/next of kin if the participants were under 18.

## Author Contributions

XL, LC, and XW designed the experimental studies. LC, WJ, HW, XL, and XW collected the experimental data. LC and JG conducted the data analysis. JG, XL, XW, LC, and WJ wrote the manuscript. All authors contributed to the article and approved the submitted version.

## Conflict of Interest

HW was employed by the Shanghai Fujia Cultural Development Co., Ltd. The remaining authors declare that the research was conducted in the absence of any commercial or financial relationships that could be construed as a potential conflict of interest.

## Publisher’s Note

All claims expressed in this article are solely those of the authors and do not necessarily represent those of their affiliated organizations, or those of the publisher, the editors and the reviewers. Any product that may be evaluated in this article, or claim that may be made by its manufacturer, is not guaranteed or endorsed by the publisher.

## Footnotes

1 The eSense Attention meter indicates the intensity of a user’s level of mental “focus” or “attention,” and the eSense Meditation meter indicates the level of a user’s mental “calmness” or “relaxation.” Their values range from 0 to 100. As shown in Figure 1, the orange and blue lines represent the eSense Attention and Meditation meter, respectively, based on algorithms that convert original EEG signals of different frequencies (alpha, beta, etc.) to the current two waves. More details on the eSense Attention and Meditation algorithms can be found on Neurosky website http://neurosky.com/biosensors/eeg-sensor/algorithms/.

2 Notably, when a cutoff score of 9 is used as the criteria for clinical depression, the percentage of depression was 22.8% (36/158) in the sample. Since this sample was from a boarding high school where students had high pressure to apply for studying abroad, we had a relatively high percentage of moderate to severe depression. Among these subjects, our brainwave model correctly identified 77.8% subjects with state depression (28 out of 36).

3 When a cutoff score of 16 is used for clinical depression, the percentage is 18.31% (13/71). Given that we purposefully recruited study participants who were suffering from various emotional disturbances and seeking psychological assistance, this percentage of depression is not unexpected. Notably, among these participants with mild or more severe depression, our EEG brainwave model correctly identified 92.3% subjects as showing symptoms of state depression (12 out of 13 subjects).
