# Supplementary material for: Research on the Method of Depression Detection by Single-Channel Electroencephalography Sensor
Source: Front Psychol. 2022 Jul 13;13:850159. doi: 10.3389/fpsyg.2022.850159 (PMC9326502; doi:10.3389/fpsyg.2022.850159)
Supplement: Supplementary file 1 [file Data_Sheet_1.docx]

**SI Appendix A: Technical Description of NeuroSky’s MindWave Mobile Headset**

**1. The TGAM module**

The TGAM module is a programmable semiconductor chip based on the ThinkGear ASIC module (Yin et al., 2020). It is the core of brainwave sensing technology inside NeuroSky’s MindWave mobile headset, which enables a device to interface with users’ brainwaves (Neurosky, 2011; Yin et al., 2020).

The TGAM module incorporates the sensor that touches the forehead, the contact and reference points in the ear clip, and the on-board chip that processes all data (Neurosky, 2011). The EEG signal-processing procedure of the TGAM module to obtain attention and meditation scores is illustrated in Figure 6 (Yin et al., 2020).

The TGAM module is embedded in the Neurosky headset, which can realize the functions of EEG signal acquisition, filtering, amplification, A/D conversion, data processing and analysis, etc (Garcia, 2018). The original EEG signal is collected at the sampling frequency of 512Hz, at the same time, the noise from muscle movement and electrical interference is filtered out, and the anti-interference ability is strong. It also includes a notch filter to eliminate 60 Hz noise from a power source. Since there are no wires attaching the electrode to an analysis device, interference due to electrode wire length is greatly reduced. Finally, the collected signal is converted into digital signal, and the communication with the computer is maintained by Bluetooth wireless transmission mode. The NeuroSky headset can sample data at up to 512 samples per second. In addition to the raw EEG data, the Mindset can output calculated delta (0.5 - 2.75Hz), theta (3.5 - 6.75Hz), low alpha (7.5 - 9.25Hz), high alpha (10 - 11.75Hz), low beta (13 - 16.75Hz), high beta (18 - 29.75Hz), low gamma (31 - 39.75Hz), and mid gamma (41 - 49.75Hz) waves as well as blink strength. It also outputs Neurosky proprietary attention and meditation signals that are meant to identify when a subject is paying attention or is relaxed. These attention and meditation signals are created using data from the other frequency bands (e.g. alpha, beta, gamma, etc).

Bluetooth module

Filter circuit

Amplifying circuit

EEG signal

ThinkGear processor

A/D conversion

Types of data output from TGAM

- eSense Attention and Meditation meter values
- EEG band power values for delta, theta, alpha, beta, and gamma

**Figure 6. EEG signal-processing procedure of the TGAM module**.

The original EEG signal is collected at a sampling frequency of 512 Hz, which can filter out various noise interferences, including extraneous noise and electrical interference, and amplify and collect the original EEG signals. Finally, the collected EEG signal is converted into a digital signal. The raw brainwaves and eSense Meters (attention and meditation) are calculated on the TGAM chip (Neurosky, 2011), and the calculated values are wirelessly sent through the Bluetooth module to a connected computer. In addition, the TGAM module has low energy consumption and adopts a serial port standard output interface. Figure 7 shows the chip principle (Zhang, et al., 2019).


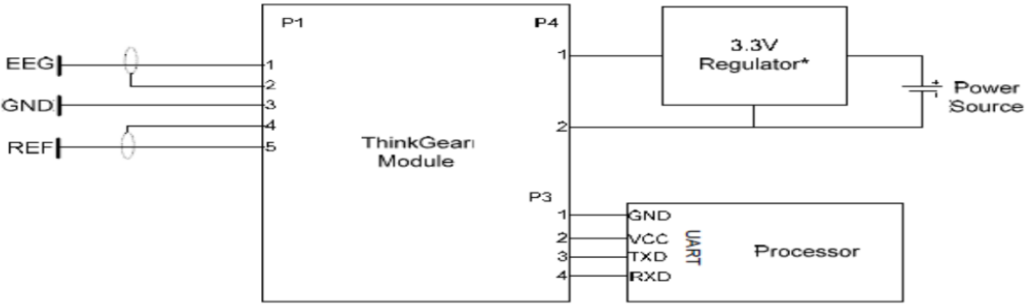


**Figure 7. Schematic of the TGAM module**.

**2. eSense Meter**

eSense is a NeuroSky's proprietary algorithm to characterize mental states (Neurosky, 2011). To calculate eSense, first, the raw brainwave signal is amplified, and the extraneous noise and muscle movement are removed by NeuroSky ThinkGear technology. Then, the eSense algorithm is applied to calculate the processed signal, leading to the interpreted eSense meter values.

The eSense meters indicate how effectively the user is engaging attention or meditation. Specifically, the eSense attention meter shows the intensity of one's level of mental “focus” or “attention”. Distractions, wandering thoughts, and anxiety may lower the level of attention meters. The eSense meditation meter shows the level of one's mental “calmness” or “relaxation”. It measures a user's mental states and is associated with reduced activity due to active mental processes in the brain. Distractions, anxiety, agitation, and sensory stimuli may decrease the level of the meditation meter. The meter value of each type of eSense is 0-100. The eSense meter value is not an exact number but describes the ranges of activity (Zhang, et al., 2019).

**Table 3. EEG Bandwidth and Corresponding Mental State and Condition**

| **Brainwave Type** | **Frequency Range** | **Mental States and Conditions** |
| --- | --- | --- |
| Delta | 0.1 Hz to 3 Hz | Deep, dreamless sleep, non-REM sleep, unconscious |
| Theta | 4 Hz to 7 Hz | Intuitive, creative, recall, fantasy, imaginary, dream |
| Alpha | 8 Hz to 12 Hz | Relaxed, but not drowsy, tranquil, conscious |
| Low Beta | 12 Hz to 15 Hz | Formerly SMR, relaxed yet focused, integrated |
| Midrange Beta | 16 Hz to 20 Hz | Thinking, aware of self & surroundings |
| High Beta | 21 Hz to 30 Hz | Alertness, agitation |

**3. The M-shaped model**

As shown in Figure 8, the EEG signal characteristics of M-shaped depressive mood were determined in the following manner (and we added these descriptions in the supplementary file).

The EEG signals of five consecutive seconds form the M-shaped EEG signal characteristics, and the values of the five consecutive seconds generated by the Meditation or Attention algorithms are D1, D2, D3, D4 and D5. Specifically, D1 represents the value of the first second of the five consecutive seconds. D2 represents the value of the second second of the five consecutive seconds and corresponds to the first peak of the M-shaped EEG signal. D3 represents the value of the third of the five consecutive seconds and corresponds to the trough of the M-shaped EEG signal. D4 represents the value of the fourth of the five consecutive seconds and corresponds to the second peak of the M-shaped EEG signal. Lastly, D5 represents the value of the fifth of the five consecutive seconds. Note that the values should follow these rules: D1<D2, D2>D3, D3<D4 and D4>D5. An M-shaped pattern is identified as long as D2-D1=D4-D5, and D2-D3=D4-D3. If the above conditions are satisfied simultaneously, the brain wave signal is characteristic of the brain wave signal of M-like depressive mood. These determination rules were developed and validated in our pilot study.


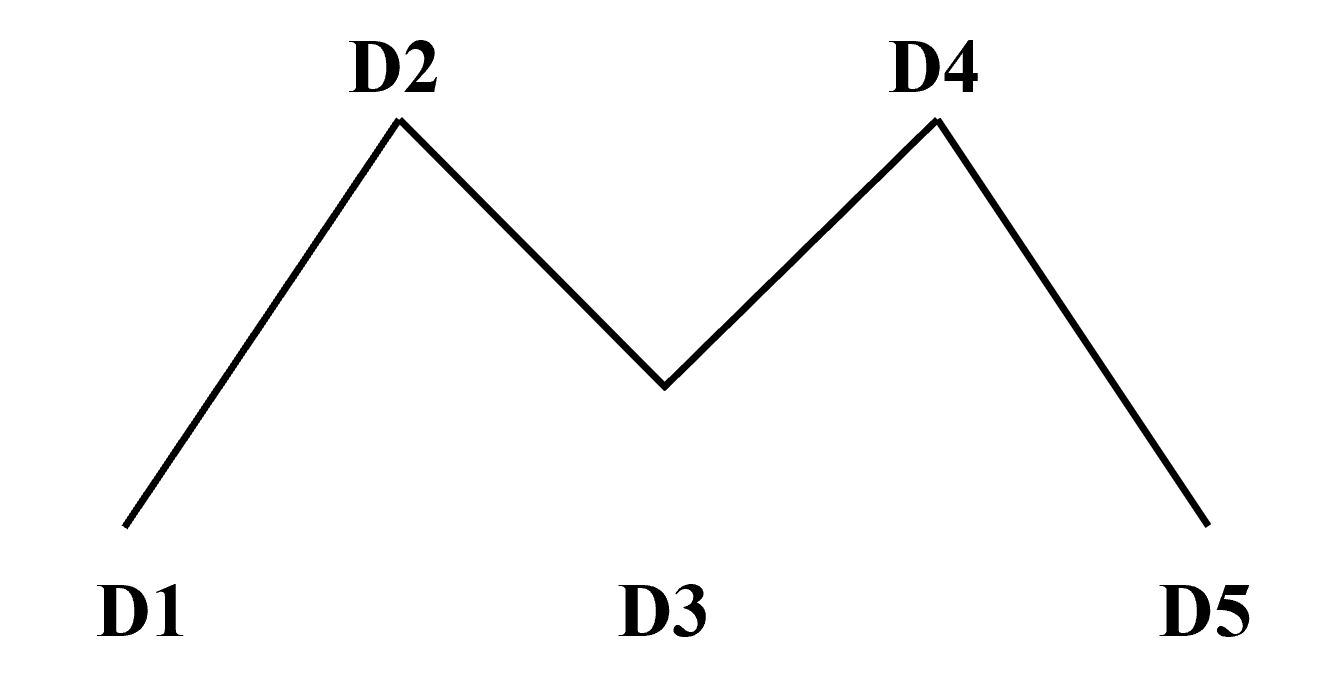


**Figure 8. The M-shaped pattern**

**SI Appendix B: Nine-Symptom Checklist of PHQ-9**

**Table 4. Nine-Symptom Checklist**

| Over the last 2 weeks, how often have you been bothered by any of the following problems? | Not at all | Several days | More than a week | Nearly every day |
| --- | --- | --- | --- | --- |
| 1.Little interest or pleasure in doing things | 0 | 1 | 2 | 3 |
| 2. Feeling down, depressed, or hopeless | 0 | 1 | 2 | 3 |
| 3. Trouble falling or staying asleep, or sleeping too much | 0 | 1 | 2 | 3 |
| 4. Feeling tired or having little energy | 0 | 1 | 2 | 3 |
| 5. Poor appetite or overeating | 0 | 1 | 2 | 3 |
| 6. Feeling bad about yourself - or that you are a failure or have let yourself or your family down | 0 | 1 | 2 | 3 |
| 7. Trouble concentrating on things, such as reading the newspaper or watching television | 0 | 1 | 2 | 3 |
| 8. Moving or speaking so slowly that other people could have noticed? Or the opposite - being so fidgety or restless that you have been moving around a lot more than usual | 0 | 1 | 2 | 3 |
| 9. Thoughts that you would be better off dead or of hurting yourself in some way | 0 | 1 | 2 | 3 |

**SI Appendix C: Items of the HAM-D**

**Table 5. Items of the HAM-D17**

| Item No. | Score Range | Symptom | Score |
| --- | --- | --- | --- |
| 1 | 0-4 | Depressed mood | 0=Absent  1=Mild  2=Moderate  3=Severe  4=Extremely severe |
| 2 | 0-4 | Guilt |  |
| 3 | 0-4 | Suicide |  |
| 4 | 0-2 | Insomnia (early) |  |
| 5 | 0-2 | Insomnia (middle) |  |
| 6 | 0-2 | Insomnia (delayed) |  |
| 7 | 0-4 | Work and interests |  |
| 8 | 0-4 | Retardation |  |
| 9 | 0-4 | Agitation |  |
| 10 | 0-4 | Psychic anxiety |  |
| 11 | 0-4 | Somatic anxiety |  |
| 12 | 0-2 | Gastrointestinal |  |
| 13 | 0-2 | General somatic |  |
| 14 | 0-2 | Loss of libido |  |
| 15 | 0-4 | Hypochondriasis |  |
| 16 | 0-2 | Loss of weight |  |
| 17 | 0-2 | Loss of insight |  |

**SI References**

Garcia, L.N., Guerreromosquera, C., Colomer, M., et al. (2018). Evoked and oscillatory EEG activity differentiates language discrimination in young monolingual and bilingual infants, *Sci. Rep*. 8 (1), 2770.

Neurosky, I. (2011). MindWave User Guide. http://developer.neurosky.com/docs/lib/exe/fetch.php?media=mindwave_user_guide_en.pdf adresinden erişilmiştir.

Yin, L., Zhang, C., and Cui, Z. (2020). Experimental research on real-time acquisition and monitoring of wearable EEG based on TGAM module. *Comput. Commun.* 151, 76-85. doi: 10.1016/j.comcom.2019.12.055

Zhang, S., Li, B., Chen, X., Lu, T., Zhao, C., Ren, J., et al. (2019). “Research on the Method of Evaluating Psychological Stress by EEG,” in *IOP Conference Series: Earth and Environmental Science* (Vol. 310, No. 4, p. 042033). IOP Publishing.
